# Dosimetric comparison of three different treatment modalities for total scalp irradiation: the conventional lateral photon–electron technique, helical tomotherapy, and volumetric-modulated arc therapy

**DOI:** 10.1093/jrr/rru049

**Published:** 2014-06-13

**Authors:** Jin Ho Song, Ji-Young Jung, Hyung-Wook Park, Gi Woong Lee, Soo-Min Chae, Chul Seung Kay, Seok Hyun SON

**Affiliations:** 1 Department of Radiation Oncology, Seoul St. Mary's hospital, College of Medicine, The Catholic University of Korea, Seoul, Korea; 2 Department of Radiation Oncology, Incheon St. Mary's hospital, College of Medicine, The Catholic University of Korea, Seoul, Korea; 3 Department of Radiation Oncology, Yeouido St. Mary's Hospital, College of Medicine, The Catholic University of Korea, Seoul, Korea; 4 Department of Radiation Oncology, Cheju Halla General Hospital, Jeju, Korea

**Keywords:** total scalp irradiation; lateral photon–electron technique; helical tomotherapy; volumetric-modulated arc therapy, hippocampus

## Abstract

The aim of this study was to compare lateral photon–electron (LPE), helical tomotherapy (HT), and volumetric-modulated arc therapy (VMAT) plans for total scalp irradiation. We selected a single adult model case and compared the dosimetric results for the three plans. All plans mainly used 6-MV photon beams, and the prescription dose was 60 Gy in 30 fractions. First, we compared the LPE, HT and VMAT plans, with all plans including a 1-cm bolus. We also compared HT plans with and without the bolus. The conformity indices for LPE, HT and VMAT were 1.73, 1.35 and 1.49, respectively. The HT plan showed the best conformity and the LPE plan showed the worst. However, the plans had similar homogeneity indexes. The dose to the hippocampus was the highest in the VMAT plan, with a mean of 6.7 Gy, compared with 3.5 Gy in the LPE plan and 4.8 Gy in the HT plan. The doses to the optical structures were all within the clinically acceptable range. The beam-on time and monitor units were highest in the HT plan. The HT plans with and without a bolus showed similar target coverage and organ-at-risk (OAR) sparing. The HT plan showed the best target coverage and conformity, with low doses to the brain and hippocampus. This plan also had the advantage of not necessarily requiring a bolus. Although the VMAT plan showed better conformity than the LPE plan and acceptable OAR sparing, the dose to the hippocampus should be considered when high doses are prescribed.

## INTRODUCTION

Total scalp irradiation is used to treat several conditions such as lymphoma, Kaposi's sarcoma, angiosarcoma, and extensive skin cancer [1–5]. Historically, homogenous scalp irradiation has presented technical and dosimetric challenges because of the complex shape and superficial nature of the target.

Several techniques have been suggested for conventional linear-accelerator treatment. Because of the relatively high surface dose provided by electrons, techniques using several electron beams have been developed [1, 6, 7]. However, several problems have been associated with the use of multiple electron beams, including dose heterogeneity across the target, numerous field junctions, and laborious treatment setup. To overcome these disadvantages, Akazawa incorporated a megavoltage photon beam and introduced the lateral photon–electron technique (LPE) [8]. This technique incorporates parallel opposing lateral photon beams, which cover the outer rind of the scalp around the top of the head, and lateral electron fields, which cover the remaining lateral portion of the scalp, matched with the photon beam. Tung *et al*. overlapped the photon and electron fields to improve dose uniformity [9]. Kinard *et al*. described a photon shell technique with four coplanar arcs [10].

With the recent advancement of radiation delivery machines, several newer modalities have been used for total scalp irradiation. Intensity-modulated radiation therapy (IMRT), high-dose-rate (HDR) brachytherapy, and serial tomotherapy have been studied by several investigators [2, 11–13]. Wojcicka *et al*. compared step-and-shoot IMRT and HDR brachytherapy with LPE, concluding that IMRT provided the best coverage and homogeneity. HDR was the most conformal plan, but the total dose delivered was limited because of the brain and optical structures [13]. Locke *et al*. compared serial tomotherapy with LPE, showing that LPE was superior because serial tomotherapy was associated with a substantial dose to the brain and optical structures [11].

Helical tomotherapy (HT) is a form of computed tomography (CT)-guided IMRT that uses 6-MV beams modulated by a 64-leaf binary multileaf collimator (MLC) [12]. HT is well suited to scalp irradiation because of its ability to deliver a tangential beam to any point on the scalp. It also does not have a field-matching problem. Another newer form of IMRT based on volumetric-modulated arc therapy (VMAT) has also become clinically available. VMAT machines can deliver radiation consisting of one or more arcs using a varying dose rate, changing gantry rotation speed, and bidirectional MLC motion [14]. VMAT also appears to be a good modality for scalp irradiation because it shares some of the advantages of HT, yet also offers a shorter treatment time. This reduced treatment time is possible because VMAT involves a cone beam, whereas HT involves a fan beam.

In this study, we selected a single adult model case for total scalp irradiation and performed a treatment-planning study to compare the HT and VMAT plans with the LPE plan, the most commonly used technique in conventional plans for total scalp irradiation. Several dosimetric parameters were compared, and we additionally focused on hippocampus sparing, which has recently been recognized as an important issue for neurocognitive function when treating the brain area [15].

## MATERIALS AND METHODS

### IRB approval

We received approval for this study from the Institutional Review Board of the Incheon St. Mary's Hospital, the Catholic University of Korea (Reference No. OC13RISI0016).

### Target delineation and prescription

We selected an adult patient without a visible gross tumor on imaging studies. A CT scan was obtained while the patient was immobilized using a thermoplastic head mask. The CT images were acquired in 2.5-mm thick slices. The target volume and normal structures were delineated by a single experienced physician.

The target was delineated on the whole scalp from the surface of the scalp skin to the depth of the cranium. The organs at risk (OARs) used for optimization included the brain, brain stem, hippocampus, eyeballs, lens, and optic nerves. The hippocampus was contoured based on the fusion of magnetic resonance imaging (MRI) scans with simulation CT scans and the contouring guideline suggested by Gondi *et al* [16]. A hippocampal avoidance region was also created using a 5-mm volumetric expansion around the hippocampus, and this OAR was used for the planning and dosimetric analyses.

The prescription dose was 60 Gy to the target in 30 fractions. The plans were optimized to achieve a target coverage with at least 90% of the planning target volume (PTV) receiving 100% of the prescription dose (V_60Gy_ > 90%) and to minimize the dose to the OARs. To achieve these planning goals, a 1-cm thick bolus was needed for the LPE and VMAT plans. It was impossible to achieve a PTV-V_60Gy_ > 90% without a bolus for the LPE and VMAT plans. However, a bolus was not essential for the HT plan to achieve the planning goal. This study employed the use of a virtual bolus created by the planning system (Eclipse ver. 8.9) rather than an actual bolus.

### The LPE, HT and VMAT treatment plans

**Fig. 1.**
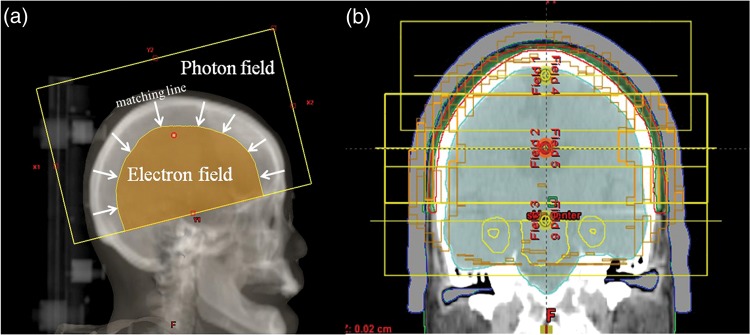
(**a**) The photon and electron field in the conventional plan. The fields are matched on the skin. The arrowheads indicate the matching line. (**b**) The positions of the three centers in the volumetric-modulated arc therapy plan.

The LPE plan consisted of opposing lateral photon and electron beams, as previously described by Akazawa [8]. The outer field comprised 6-MV photon beams with a field size of 22 × 14 cm^2^. The inner field comprised 9-MeV electron beams with a cone size of 25 cm. The electron beam was matched to the photon field on the skin (Fig. 1a). For the LPE plan, a 1-cm thick virtual bolus was applied. A pencil-beam algorithm was used for the photon field calculations, and an electron Monte Carlo algorithm was used for the electron field, as provided by the Eclipse ver. 8.9 treatment-planning system. The calculation grid was 2.5 × 2.5 mm.

The Tomotherapy Hi-Art system (Tomotherapy Inc., Madison, WI, USA) is an IMRT modality in which a 6-MV linear accelerator is mounted on a ring gantry that continuously rotates during treatment. The couch simultaneously moves through the rotating beam plane so that the radiation is delivered in a helical fashion [17]. For HT planning, three parameters should be selected: the field width (the slice thickness of the radiation field projected at the isocenter along the gantry rotation axis) was set to 1.0 cm, the pitch (the couch movement relative to the field width during one gantry rotation) was set to 0.172, and the modulation factor (the ratio between the maximum number of opening leaves and the average number of opening leaves in active gantry rotation) was set to 3.20. The bolus was not essential for HT planning. Accordingly, we generated two plans: a plan with the bolus and a plan without the bolus. Tomotherapy Planning Station ver. 4.0.4 was used, and the calculation grid was 1.95 × 1.95 mm.

RapidArc (Varian Medical Systems, Palo Alto, CA, USA) treatment was planned with the Eclipse ver. 10.0.42 planning system. The plans were optimized using the Progressive Resolution Optimizer (PRO) ver. 10.0.28 and calculated with the Anisotropic Analytical Algorithm (AAA) ver. 10.0.28. It was impossible to achieve proper target coverage with single- or double-isocenter plans. Therefore, the final approved plan consisted of three centers that were located 4 cm apart. The locations of the three centers are shown in Fig. 1b. A total of nine 6-MV clockwise 358° arcs (181° to 179°) were used with no treatment table rotation. The collimator angles were set to 90° for each arc, and the MLC margin was 0.5 cm from the target. For the VMAT plan, a 1-cm thick bolus was used. The calculation grid was 2.5 × 2.5 mm.

### Analysis and comparisons between plans

Plan conformity and homogeneity were assessed using the following parameters.

#### Conformity index

The conformity index (CI) is a ratio used to evaluate how well the target fits the prescription isodose volume in the plan [18]:

$$CI=\frac{V_{PTV}}{TV_{PV}}\times \frac{V_{TV}}{TV_{PV}},$$

where V_PTV_ = PTV volume, V_TV_ = treatment volume of the prescribed isodose lines, TV_PV_ = volume of V_PTV_ within the V_TV_. A lower CI value indicates better conformity.

#### Homogeneity index

The homogeneity index (HI) is a ratio used to evaluate dose homogeneity in the PTV [19, 20]:

$$HI=\frac{D_{1\%}}{D_{99\%}},$$

where D1% and D99% are the minimum doses delivered to 1% and 99% of the PTV. A lower HI value indicates better homogeneity.

#### Conformation number

The conformation number (CN) is an index that incorporates both target coverage and the extent to which the normal tissues are spared. CN was introduced by vant't Riet *et al* [21].

$$CN=\frac{TV_{RI}}{TV}\times \frac{TV_{RI}}{V_{RI}},$$

where TV = target volume, TV_RI_ = target volume covered by the reference dose, and V_RI_ = volume of the reference dose.

#### Conformation index

The conformation index (COIN) is an index that was proposed by Baltas *et al*. and is computed for a reference dose [22]:

$$COIN=CN\times \prod_{i=1}^{N_{CO}}\left[1-\frac{V_{COref,i}}{V_{CO,i}}\right],$$

where CN = conformation number, N_CO_ = number of critical organs, V_COref,i_ = critical volume receiving at least the reference dose, and V_CO,i_ = critical organ volume.

#### ‘Index A’ improvement ratio

The ‘Index A’ improvement ratio (ΔIndexA(%)) is a ratio used to evaluate the improvement of ‘Index A’ between the plans. It was employed for the CI, HI, CN and COIN indices [19]:

$$\Delta \;Index\;A\;(\%)=\frac{Index\;A_{\;plan2}-Index\;A_{\;plan1}}{Index\;A_{\;plan1}}\times 100\%.$$

#### Quality index

The quality index (QI) is an index used to evaluate differences in the absorbed doses at the OARs. This index uses the maximum dose for serial OARs (optic nerves in our study) and the mean dose for parallel OARs (all other OARs in our study) [20].

$$QI_{Serial}=\frac{D_{max}^{\;plan1}}{D_{max}^{\;plan2}},\;QI_{Parallel}=\frac{D_{mean}^{\;plan1}}{D_{mean}^{\;plan2}}.$$

We compared the LPE, HT and VMAT plans by assessing these parameters, which were all planned with a 1-cm thick bolus. The beam-on time and monitor units (MUs) of each system were also compared. Because an optimal HT plan could be obtained without the use of a bolus, we also compared HT plans with and without the bolus.

## RESULTS

### Comparisons of the LPE, HT and VMAT plans with a bolus

**Fig. 2.**
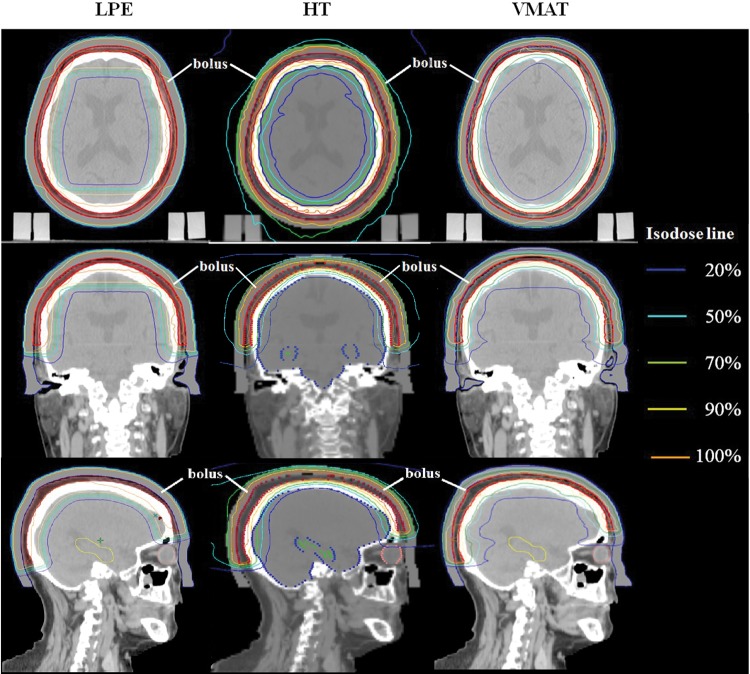
Axial, coronal and sagittal images of the isodose distribution of each plan with a bolus. (LPE = lateral photon–electron plan, HT = helical tomotherapy plan, VMAT = volumetric-modulated arc therapy plan.)

**Fig. 3.**
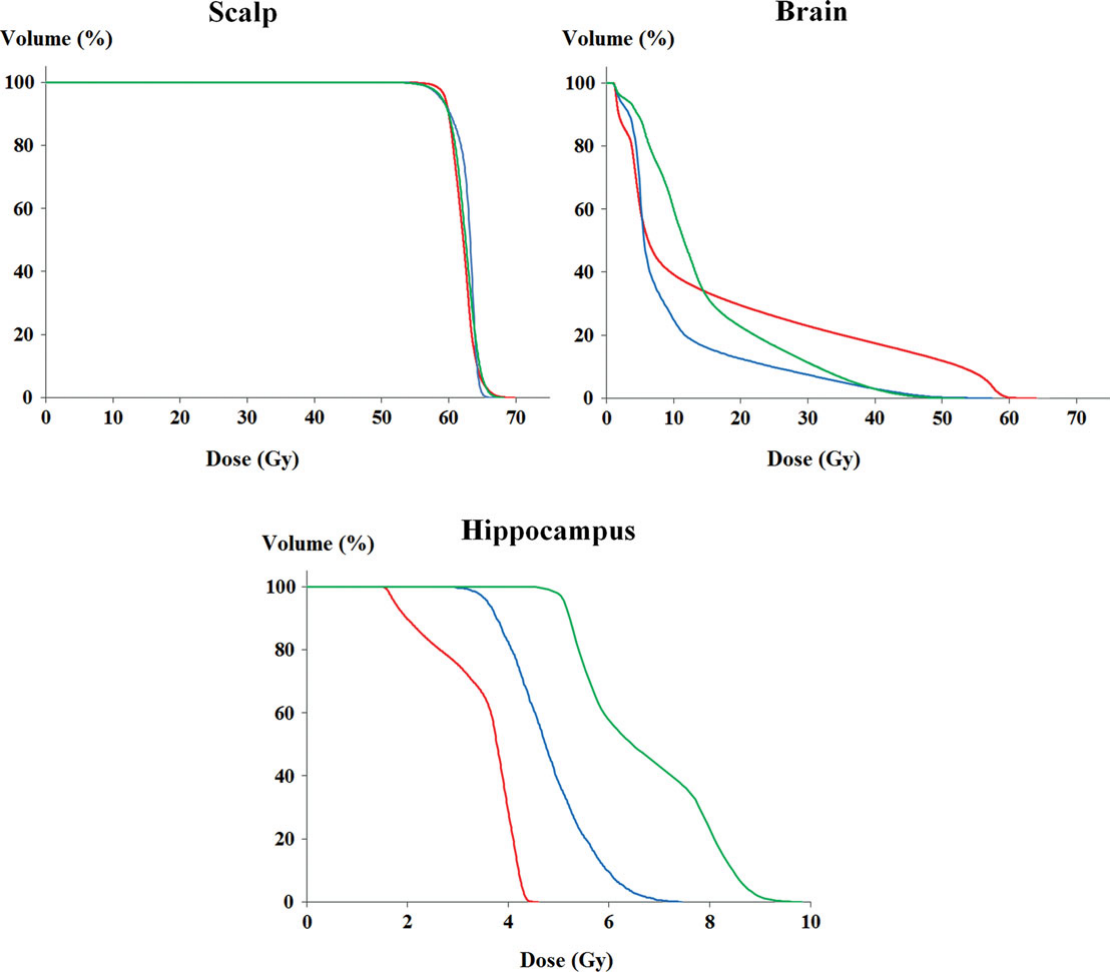
Dose–volume histograms for the scalp, brain and hippocampus. (Red line = the conventional lateral photon–electron plan, blue line = helical tomotherapy, green line = volumetric-modulated arc therapy.)

Isodose curves and the dose–volume histograms (DVHs) of the target are shown in Figs 2 and 3. Table 1 lists the indices that indicate the conformity and homogeneity of the target coverage in each plan. The CI was 1.73 with LPE, 1.35 with HT, and 1.49 with VMAT, indicating that the HT plan had the best conformity and the LPE plan had the worst. The CI was improved by 22.0% in the HT plan and 13.9% in the VMAT plan compared with the LPE plan. The HT plan showed 10.4% better CI compared with the VMAT plan. While the CI clearly differed between the plans, the HI was similar across plans. Homogeneity was poorest for the VMAT plan; however, the difference was only 1.7%.

**Table 1.** Dosimetric comparisons of target coverage in the LPE, HT and VMAT plans

	LPE	HT	VMAT		HT/LPE	VMAT/LPE	VMAT/HT
CI	1.73	1.35	1.49	ΔCI(%)	−22.0	−13.9	10.4
HI	1.15	1.15	1.17	ΔHI(%)	0	1.7	1.7
CN	0.57	0.74	0.66	ΔCN(%)	29.8	15.8	−10.8
COIN	0.56	0.74	0.66	ΔCOIN(%)	32.1	17.9	−10.8

LPE = lateral photon–electron plan, HT = helical tomotherapy, VMAT = volumetric-modulated arc therapy, CI = conformity index, HI = homogeneity index, CN = conformation number, COIN = conformation index.

The CN and COIN results resembled the CI results. The CN and COIN were highest in the HT plan, second highest in the VMAT plan and lowest in the LPE plan. These findings suggest that the HT plan had the best conformity and the smallest volume of normal structures included in the high-dose area. The difference between the LPE and HT plans was > 25%, that between the LPE and VMAT plans was ∼17%, and that between the HT and VMAT plans was 10.8%.

Dosimetric comparisons of the OARs are shown in Tables 2 and 3. The LPE plan featured the highest mean and maximum dose to the brain, as well as the largest brain volumes that received at least 20 Gy, 30 Gy, 40 Gy and 50 Gy (V_20Gy,_ V_30Gy_, V_40Gy_ and V_50Gy_). However, as shown in Fig. 3, the DVHs for LPE and VMAT crossed at the 15-Gy level, indicating that the volume receiving < 15 Gy was larger in the VMAT plan.

**Table 2.** Dosimetric analyses of the mean, maximum and minimum doses to nine OARs in the LPE, HT and VMAT plans

OARs	Mean (Gy)			Max (Gy)			Min (Gy)		
	LPE	HT	VMAT	LPE	HT	VMAT	LPE	HT	VMAT
Brain	16.8	9.8	14.6	64.0	57.4	53.1	0.9	1.0	0.9
Brain stem	2.5	3.6	6.2	4.0	5.7	8.6	1.3	1.5	2.7
Hippocampus	3.5	4.8	6.7	4.6	6.5	9.8	1.5	4.7	4.4
Lt eyeball	1.6	5.2	6.2	1.8	18.7	13.3	1.4	1.8	3.9
Rt eyeball	2.0	4.5	5.9	6.2	17.3	12.2	1.2	1.7	3.8
Lt lens	1.6	3.0	6.0	1.8	3.6	6.2	1.4	2.4	5.5
Rt lens	1.7	2.4	5.5	2.0	3.0	6.4	1.5	2.0	4.5
Lt optic nerve	1.9	3.5	5.9	2.3	5.0	6.2	1.7	2.8	5.4
Rt optic nerve	1.8	2.9	6.4	2.1	3.6	6.6	1.6	2.4	5.9
	V_10Gy_ (%)			V_20Gy_ (%)			V_30Gy_ (%)		
Brain	39.2	25.0	48.9	29.4	12.5	22.6	22.9	7.4	11.3
	V_40Gy_ (%)			V_50Gy_ (%)					
Brain	17.4	3.0	2.9	11.8	0.3	0.1			

OARs = organs at risk, LPE = lateral photon–electron plan, HT = helical tomotherapy, VMAT = volumetric-modulated arc therapy, Lt = left side, Rt = right side, V_10Gy_ = volume receiving at least 10 Gy, V_20Gy_ = volume receiving at least 20 Gy, V_30Gy_ = volume receiving at least 30 Gy, V_40Gy_ = volume receiving at least 40 Gy, V_50Gy_ = volume receiving at least 50 Gy.

**Table 3.** Dosimetric comparisons of the QIs for nine OARs between the LPE, HT and VMAT plans

OARs	QI values		
	HT/LPE	VMAT/LPE	VMAT/HT
Brain	0.58	0.86	1.48
Brain stem	1.44	2.48	1.72
Hippocampus	1.37	1.91	1.39
Lt eyeball	3.25	3.87	1.19
Rt Eyeball	2.25	2.96	1.31
Lt lens	1.87	3.44	2.00
Rt Lens	1.41	3.23	2.29
Lt optic nerve	2.17	2.69	1.24
Rt optic nerve	1.74	3.14	1.83
mean ± SD	1.78 ± 0.74	2.73 ± 0.90	1.60 ± 0.38

OARs = organs at risk, QI = quality index, LPE = lateral photon–electron plan, HT = helical tomotherapy, VMAT = volumetric-modulated arc therapy, Lt = left side, Rt = right side, SD = standard deviation.

Other normal structures, including the hippocampus, received the lowest mean and maximum doses in the LPE plan (Table 2, Fig. 3). However, all doses to normal structures were within clinically acceptable levels in the HT and VMAT plans. The mean dose to the hippocampus was 3.5 Gy in the LPE plan, 4.8 Gy in the HT plan and 6.7 Gy in the VMAT plan.

Comparing the QI values of the LPE plan with the others, the QI values of all the OARs except for the brain were over 1.0. This result indicates that all OARs except the brain received a lower dose in the LPE plan than they did in the other plans. The mean QI values were 1.78 ± 0.74 for the HT versus LPE plan, and 2.73 ± 0.90 for the VMAT versus LPE plan. However, the respective brain QI values were 0.58 and 0.86.

The QI values showed that all nine OARs received lower doses in the HT plan compared with the VMAT plan. The mean QI value for VMAT versus HT was 1.60 ± 0.38.

The beam-on times and MUs for a single fraction (to deliver 2 Gy to the PTV) are listed in Table 4. The LPE plan had the shortest beam-on time. The beam-on times were 1.5 min for the LPE plan, 14.7 min for the HT plan and 11.3 min for the VMAT plan. The MUs were also the lowest in the LPE plan (616). The MUs for the HT plan were 13 088, which was much higher than the MUs of 1791 in the VMAT plan. HT and VMAT differed much more substantially in terms of MUs than they did in terms of beam-on time.

**Table 4.** Beam-on times and MUs in a single fraction in the LPE, HT and VMAT plans

	LPE	HT	VMAT
Beam-on time (min)	1.5	14.7	11.3
MUs	616	13 088	1 791

LPE = lateral photon–electron plan, HT = helical tomotherapy, VMAT = volumetric-modulated arc therapy, MUs = Monitor Units.

### Comparisons of HT plans with and without a bolus

For LPE and VMAT, it was impossible to obtain acceptable plans that satisfied the planning goal without using a bolus. However, we were able to obtain a well-designed HT plan without a bolus. Tables 5 and 6 present the target and OAR analyses for the HT plans with and without a bolus. The CI, HI, CN and COIN were similar between the plans. The CI, CN and COIN were 6.7% better in the HT plan without the bolus, suggesting better conformity. The HI was also 0.8% better in the HT plan without the bolus.

**Table 5.** Dosimetric comparisons of target coverage for HT plans with and without a bolus

	HT with a bolus	HT without a bolus		HT without a bolus/HT with a bolus
CI	1.35	1.26	ΔCI (%)	−6.7
HI	1.15	1.14	ΔHI (%)	−0.8
CN	0.74	0.79	ΔCN (%)	6.7
COIN	0.74	0.79	ΔCOIN (%)	6.7

HT = helical tomotherapy, CI = conformity index, HI = homogeneity index, CN = conformation number, COIN = conformation index.

**Table 6.** Dosimetric comparisons of the mean, maximum and minimum doses to nine OARs for HT plans with and without a bolus

OARs	Mean (Gy)		Max (Gy)		Min (Gy)		QI value
	With a bolus	Without a bolus	With a bolus	Without a bolus	With a bolus	Without a bolus	
Brain	9.8	10.3	57.4	59.5	1.0	0.9	1.05
Brain stem	3.6	3.3	5.7	4.4	1.5	1.4	0.92
Hippocampus	4.8	4.7	6.5	6.4	4.7	3.5	0.98
Lt eyeball	5.2	4.8	18.7	20.9	1.8	2.1	0.92
Rt eyeball	4.5	6.0	17.3	19.7	1.7	1.7	1.33
Lt lens	3.0	3.7	3.6	4.5	2.4	3.1	1.23
Rt lens	2.4	2.7	3.0	3.4	2.0	2.2	1.12
Lt optic nerve	3.5	3.8	5.0	5.8	2.8	3.3	1.08
Rt optic nerve	2.9	3.2	3.6	3.9	2.4	2.7	1.10
	V_10Gy_ (%)		V_20Gy_ (%)		V_30Gy_ (%)		
Brain	25.0	25.4	12.5	13.9	7.4	8.8	
	V_40Gy_ (%)		V_50Gy_ (%)				
Brain	3.0	4.4	0.3	1.1			

OARs = organs at risk, QI = quality index, Lt = left side, Rt = right side, V_10Gy_ = volume receiving at least 10 Gy, V_20Gy_ = volume receiving at least 20 Gy, V_30Gy_ = volume receiving at least 30 Gy, V_40Gy_ = volume receiving at least 40 Gy, V_50Gy_ = volume receiving at least 50 Gy.

**Fig. 4.**
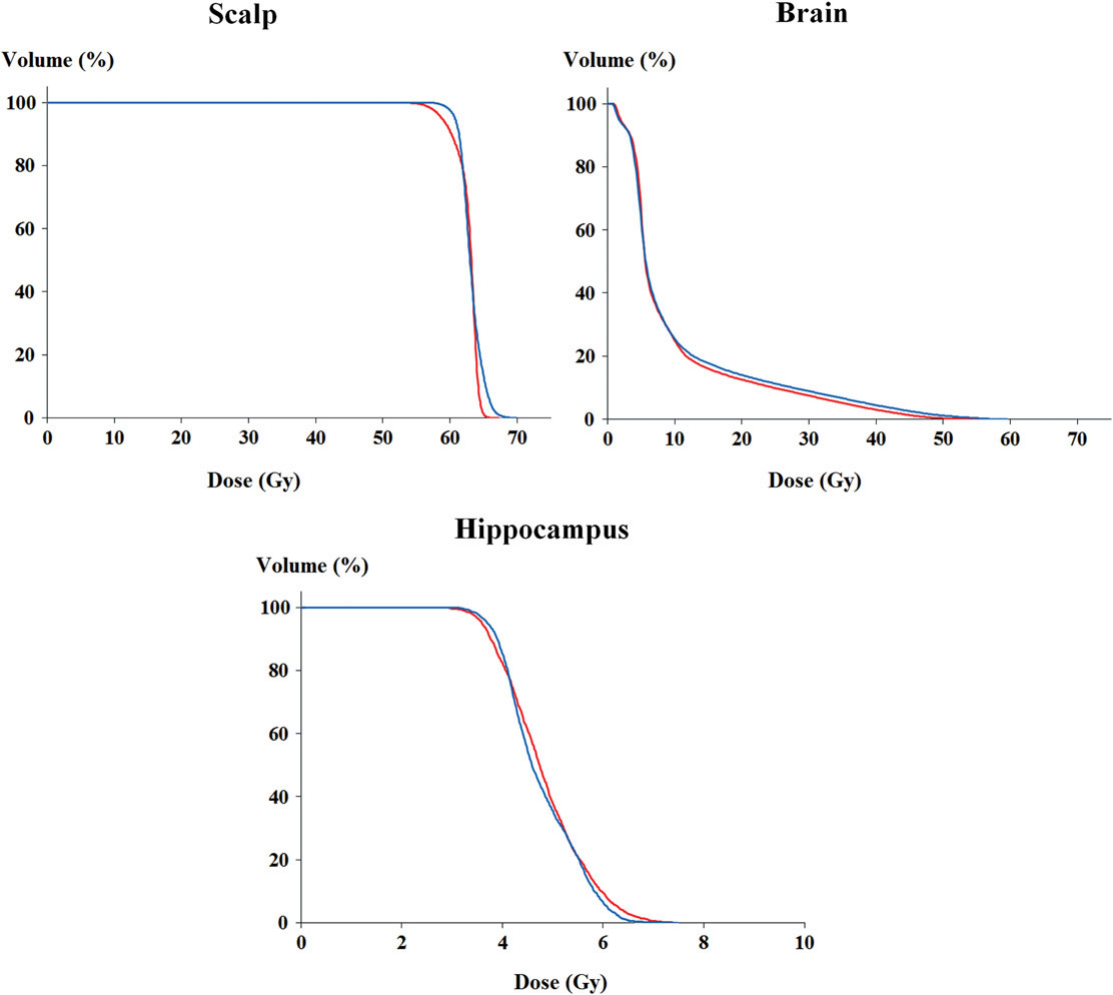
Comparisons of dose–volume histograms for the brain: helical tomotherapy plans with and without a bolus. (Red line = with a bolus; blue line = without a bolus.)

With respect to OAR sparing, the mean QI value was 1.08 ± 0.14, which suggested slightly better OAR sparing in the HT plan with the bolus. While the brain stem, left eyeball and hippocampus received a slightly lower dose in the plan without the bolus, the mean dose differences were only 0.3 Gy in the brain stem and 0.1 Gy in the hippocampus. Although the doses to normal structures were all in the clinically acceptable range in both plans, the DVHs showed that the brain volume was lower for all dose ranges (V_10Gy_, V_20Gy_, V_30Gy_, V_40Gy_ and V_50Gy_) in the plan with the bolus (Fig. 4). However, this only amounted to a 0.5 Gy difference from the mean brain dose.

## DISCUSSION

Total scalp irradiation is relatively rare in clinical practice. However, there are several diseases for which total scalp irradiation is indicated, including lymphoma, mycosis fungoides, Kaposi's sarcoma, angiosarcoma, and extensive skin cancer [1–5]. The prescription dose is variable, depending on the disease category and the purpose of radiation treatment. For example, 20–30 Gy is usually prescribed for mycosis fungoides and other lymphomas, 25–40 Gy for Kaposi's sarcoma, and 50–60 Gy for skin cancer and angiosarcoma. This dose variability and the complex shape of the scalp make it difficult to select a single best technique for all patients. In this study, a detailed dosimetric analysis was performed to compare three different techniques (the conventional LPE plan, an HT plan, and a VMAT plan) for an assumed prescription dose of 60 Gy to the scalp.

The most commonly used technique is LPE, which was first described by Akazawa [8]. From our results, the advantage of this technique over the other two techniques was that the optical structures received the lowest dose. The mean doses to several optical structures were all below 2 Gy. However, the mean brain dose was higher than for the HT and VMAT plans. In addition to the highest mean brain dose, LPE was also associated with the highest V_20Gy_, V_30Gy_, V_40Gy_ and V_50Gy._ With respect to target coverage, the LPE plan was the least conformal, although the homogeneity was similar across plans. The technical difficulty of matching the photon–electron beams was found to be another disadvantage of LPE.

The VMAT plan showed better dose conformity than the LPE plan; the conformity increased by 13.9% compared with LPE. However, the doses to the OARs were the highest in this plan, with the exception of the brain dose. The mean QI values were 2.73 compared with LPE, and 1.6 compared with HT. Nevertheless, the doses to the optical structures were within the clinically acceptable range. The mean brain dose was higher than that of HT, but lower than that of LPE.

The HT plan showed the best conformity among the plans. The conformity was increased by 10.4% compared with VMAT and 22.0% compared with LPE. The mean dose to the brain was also the lowest. Other optical structure doses were higher than those of LPE, but lower than those of VMAT. This superiority of HT compared with LPE was consistent with other studies. Orton *et al*. compared the HT plan with two conventional linear accelerator plans (LPE and concentric electron fields technique) [12]. They prescribed 40 Gy in 20 fractions, and found that the HT plan delivered a more uniform dose to the scalp and reduced the V_30Gy_ of the brain by as much as two-thirds.

Another advantage of the HT plan was that the bolus was not an essential component for planning. Unlike the LPE and VMAT plans, in which it was impossible to achieve appropriate target coverage without a bolus, the HT plans with and without the bolus did not differ markedly. It is also practically difficult to apply the bolus tightly around the entire scalp. The advantage of the bolus in the HT plan was only a slight decrease in the brain dose. The mean dose decreased by only 0.5 Gy (from 10.3 to 9.8 Gy) when 60 Gy was prescribed to the scalp. Several studies have shown prescribed doses can be delivered with HT, even to regions close to the surface, without the use of a bolus [17, 23–25]. Although the results were variable across these studies, the measured surface doses were not that different from the calculated doses, and this can be explained by the high number of tangential beams, which is unique to HT [17].

On the other hand, for cases in which the skin surface does not need to receive therapeutic doses, the VMAT plan can also be a good option. Kelly *et al*. compared static IMRT with VMAT for total dural irradiation [26]. They cropped 3 mm of the PTV to allow skin sparing, and concluded that VMAT can provide a clinically acceptable treatment plan without the use of a bolus.

However, the HT plan had the longest beam-on time and the highest MUs. The HT beam-on time was 9.8 and 1.3 times longer than the beam-on times of the LPE and VMAT plans, respectively. Although the HT plan showed only a slightly longer beam-on time than the VMAT plan, the MUs were 21.2 and 7.3 times higher than the MUs of the LPE and VMAT plans, respectively. Treatments with longer delivery times generally have the potential disadvantage of intrafraction patient motion, and higher total MU delivery may result in higher patient exposure to leakage radiation [27]. This concern regarding HT is common for other treatment sites [27, 28].

Clinically, the main concern while treating the scalp is the dose to the brain. The maximal dose to the brain was higher than 60 Gy only in the LPE plan. The brain dose was not high enough to cause brain necrosis in the HT or VMAT plans. However, neurocognitive function could be affected, even at low doses [29]. Recently, several clinical studies have suggested that radiation damage to the hippocampus plays a considerable role in neurocognitive function decline [15]. Deficits in learning, memory, and spatial processing are thought to be related to hippocampus injury in patients who have received whole brain radiation. However, a widely accepted dose guideline does not yet exist. Gondi *et al*. suggests that irradiation of 40% of the bilateral hippocampus at a dose of > 7.3 Gy is associated with long-term impairment after benign and low-grade adult brain tumor treatment [30]. Hsu *et al*. and Gutiérrez *et al*. studied the feasibility of VMAT and HT for the hippocampal sparing whole brain radiotherapy with a simultaneous integrated boost. The dose constraint used for the hippocampus was 6 Gy in both studies [31, 32]_._ In another study, Gondi *et al*. suggests that, when whole brain radiotherapy is delivered with 30 Gy in 10 fractions, it is able to reduce (i) the mean dose to the hippocampus by 5.5 Gy using HT and by 7.8 Gy using LINAC-based IMRT, and (ii) the maximum dose to 12.8 Gy using HT and to 15.3 Gy using LINAC-based IMRT [15]. In the Radiation Therapy Oncology Group (RTOG) 0933 Phase II trial for hippocampus sparing in brain metastasis treatment, the guidelines are as follows: (i) dose to 100% of the hippocampal volume (D_100_) ≤ 10 Gy and (ii) maximum dose ≤17 Gy. In our study, the VMAT plan showed the highest dose to the hippocampus, with a mean dose of 6.7 Gy (as compared with 3.5 Gy for LPE and 4.8 Gy for HT). The maximum dose was 9.8 Gy in the VMAT plan, which was higher than the 4.6 Gy dose for LPE and the 6.5 Gy dose for HT. The doses were smaller than those noted in previous studies. However, considering the hypothesis that active neural stem cells in the hippocampal dentate gyrus play a major role in neurocognitive function and that these cells are quite sensitive to radiation [29], lowering the dose to the hippocampus would be needed.

In conclusion, the results of our study showed that HT was the best modality for total scalp irradiation. The HT plan showed the best target coverage and conformity, with low doses to the brain and hippocampus. It also has the advantage of not requiring a bolus. Although the VMAT plan showed acceptable OAR sparing and better conformity compared with the LPE plan, the dose to the hippocampus should be considered when high doses ( > 60 Gy) are needed. The LPE plan has the advantage of lowering the dose to the optical structures. However, considering the LPE's conformity limitations, the large volume of the brain that received high doses, and the technical difficulty associated with matching the fields, it seems as though LPE should cede its throne to other more recent modalities.

## FUNDING

The authors wish to acknowledge the financial support of the Catholic Medical Center Research Foundation made in the program year of 2014 (5-2013-B0001-00243).
